# Questionnaire Survey-Based Quantitative Assessment of the Impact of Transitional Care on Self-Management of Patients with Acute Exacerbation of Chronic Obstructive Pulmonary Disease

**DOI:** 10.1155/2021/3634548

**Published:** 2021-11-13

**Authors:** Wenjie Xu, Hui Hu, Yanjun Mao

**Affiliations:** ^1^Department of Respiratory and Critical Care Medicine No. 1, Shanghai Pulmonary Hospital, China; ^2^Nursing Department, Shanghai Pulmonary Hospital, China

## Abstract

**Methods:**

Clinical information of 78 COPD patients treated with TC (intervention group) or routine care (control group) in Shanghai Pulmonary Hospital during March 2019 and August 2020 was gathered. Patients were followed up for 3 months after discharge. The intervention group (*n* = 39) was subjected to a TC plan for 3 months to help patients and their family caregivers for self-management of COPD. TC was provided by specially trained nurses, and patients were supported by standardized tools. Nursing measures in the control group (*n* = 79) included transitional support for 30 d after hospital discharge. In this way, patients were guaranteed to follow discharge plans and transit to outpatient nursing. Patient's anxiety and depression symptoms, sleep quality, survival quality, mobility, and life quality at admission and after 3 months of discharge were assessed by Hospital Anxiety and Depression Scale, Pittsburgh Sleep Quality Index, Quality of Life Scale Abbreviated Version, Activity of Daily Life Scale, St. George's Respiratory Questionnaire, and COPD Assessment Test.

**Results:**

Except for anxiety and depression, patient's sleep quality, survival quality, mobility, and life quality in two groups were significantly improved. Moreover, average change of total CAT score during 3 months of intervention was -5.44, while that in the control group was -1.74 (*p* = 0.011). Improvement of survival quality of patients in the intervention group (*p* = 0.001) was markedly greater than that in the control group (*p* = 0.016).

**Conclusion:**

Altogether, TC based on quantification by questionnaire survey is beneficial to COPD patient's life quality and self-management.

## 1. Introduction

Chronic respiratory diseases (CRD) are prime causes of global disability and death [1]. Chronic obstructive pulmonary disease (COPD) falls under the umbrella of CRD and is the most prevalent type. There were approximately 251 million COPD cases in the world in 2015, and this disease is expected to be the third factor of deaths in 2030 [2]. Common symptoms of COPD are shortness of breath, coughing, expectoration, or even eosinophilic airway inflammation [3]. Even if COPD can be preventable and treatable [4], resultant lower life quality and secondary psychological problems still exist including depression, anxiety, and dyspnea and impose heavy burden to patients, physically and mentally [5]. Earlier investigations suggested factors for pathogenesis of COPD, such as smoking, air pollution, infection, and vocation. Admission due to acute exacerbation of chronic obstructive pulmonary disease (AECOPD) is a grievous burden for individuals, like muscular dysfunction, dyspnea, and unable to move [6]. We devoted to developing effective nursing methods of releasing patient's negative emotions and increasing their life quality.

Transitional care (TC), also called continuous care or transitional nursing, is an extension of hospital care to guarantee the continuity of professional medical care for patients after discharge [7]. The purpose of TC is to increase patient's disease self-management, promote medical stability, and decline readmission rate via telephone follow-up, home visiting, and messages. TC has been proved to improve the prognosis of patients with chronic diseases [8, 9]. Moreover, TC can reduce medical costs by lowering emergency attendance rates and readmission rate [10]. An investigation indicated that nurses-led TC shows a positive impact on blood glucose control and treatment compliance, and lowers medical cost for diabetic patients after discharge [11]. A meta-analysis confirmed that TC significantly declines hospital readmission and mortality rate in COPD subjects [12]. For patients awaiting coronary artery bypass graft (CABG), TC can reduce anxiety, depression, and hospital times while enhancing cardiac self-efficacy and satisfaction with care [13].

In the above context, we conducted a retrospective analysis to quantitatively assess the impacts of TC on COPD patient's self-management based on a questionnaire survey. TC offers a rationale for the subsequent care of patients with COPD.

## 2. Data and Methods

### 2.1. Patient Inclusion

From March 2019 to August 2020, 79 COPD patients were treated in Shanghai Pulmonary Hospital Department of Respiratory and Critical Care Medicine No. 1 due to acute attack. They were diagnosed with COPD in accordance with Guideline for Diagnosis and Treatment of Chronic Obstructive Pulmonary Disease. All of the patients were older than 18. A patient who died during treatment was excluded, and 78 patients were finally included. The included patients were classified into the intervention group and control group (39 cases each). This study has been approved by the medical ethic committee of our hospital. Patient's information was used for research purpose only.

### 2.2. Intervention Group

Primary nurse was in charge of patient's care and evaluation from admission to follow-up and adopted one-to-one health education, including (I) respiratory muscle function exercise. (i) Pursed-lip breathing: the patient may be in the sitting, standing, or decubitus position. Patients are inhaling through their noses and exhaling through narrow mouths slowly with lips in the shape of a fish's mouth. The size of the labial contraction is adjusted by patient's own choice. The ratio of inhalation to exhalation is 1 : 2. The action is practiced twice with each time 5 min or as patient's abilities. (ii) Abdominal breathing: the patients take a comfortable position, and the whole body is relaxed. The abdomen is relaxed and bulged as inhaling, while it is contracted and sagged as exhaling. When exhaling, patients can press under ribs and on abdomen with their hands to promote abdominal muscles to contract and exhale. Abdominal breathing is practiced twice a day with each time 10 min or as their abilities. (iii) Respiratory exercise: collective respiratory rehabilitation exercises are carried out in the ward every morning. According to patient's situation, the exercise should be done step by step and acted according to their abilities. During hospitalization, patients are taught to master respiratory exercise. Atlas is distributed in convenience of their learning at home if they forget how to do it. (iv) Home oxygen therapy: patients are guided to take oxygen for no less than 15 h every day. It is advisable to take oxygen at night and before oxygen consumption is increased, such as meals and exercise. Oxygen uptake concentration is 1-2 L/min. (v) Nutrition guidance: it is advisable for patients to uptake foods with high protein, high fiber, moderate fat, moderate minerals and vitamins, low carbohydrate, low sugar, and low salt and drink more water, with frequent small meals. If significant hypoxia happens, oxygen therapy is recommended before or after a meal. (vi) Mental support: patiently listen to the patient and the difficulties they are encountering. Objectively analyze patient's state and treatment. Care, support, and encourage patients. Share successful cases with patients to build their confidence to overcome the disease. Guide watchers to encourage patients to do things according to their abilities in daily life. Increase patient's self-care ability and strengthen their role in daily life. Give guidance to watchers if they encounter difficulties during care. (vii) Telephone or WeChat follow-up was performed at the first and third months after discharge. Conduct care assessment and targeted health guidance for patients. The content includes signs and symptoms of COPD, complications or new symptoms, psychology, sleeping, home rehabilitation training, healthy behaviors, and environment. Aspect: (i) guide patients with correct and effective methods of coughing and hawking. Inform patients of self-monitoring symptoms and seeking medical advice in time if they feel uncomfortable. (ii) Evaluate whether patients participate in social activities and whether they are in a good mood. Encourage patients to actively participate in social activities. Timely psychological counseling shall be given to patients who are in an abnormal mood. (iii) Home rehabilitation training: evaluate whether patients exercise according to the discharge nursing prescription. Educate, coach, and follow up skills on the causes of poor compliance. Negotiate with patients to adjust nursing prescriptions. (iv) Healthy behaviors: evaluate whether patients take drugs, uptake oxygen, and do pursed-lip breathing daily according to prescription. Assess their sleeping and diet conditions. Help patients establish a good lifestyle and correct healthy behaviors via health education and guidance. (v) Evaluate whether patients improve risk factors of home environment. If not, guide and supervise them to improve these factors. (vi) On-site examine the inhalation methods of various inhalers through respiratory chronic disease management nursing clinic, such as salmeterol xinafoate and fluticasone propionate powder for inhalation, tiotropium bromide powder for inhalation, and budesonide formoterol powder inhaler. Test patients who need to perform pursed-lip breathing, abdominal breathing, or breathing exercises. Evaluate patient's mastery and give correct guidance to increase their self-care ability.

### 2.3. Control Group

Patients in the control group only received routine care. COPD education curriculum was performed every two weeks for 1 month, including discharge process guidance, disease factor information, pulmonary rehabilitation, and home oxygen therapy.

### 2.4. Evaluation Index

Patient's anxiety and depression symptoms, sleep quality, survival quality, mobility, and life quality at admission and after 3 months of discharge were assessed by Hospital Anxiety and Depression Scale (HADS), Pittsburgh Sleep Quality Index (PSQI), Quality of Life Scale Abbreviated Version (QOL-BREF), Activity of Daily Life Scale (Barthel index), St. George's Respiratory Questionnaire (SGRQ), and COPD Assessment Test (CAT) [14]. Details were provided in the Supplementary material.

HADS [15] was employed to assess the anxiety and depression of the subjects in this investigation. This scale contains 7 HADS-anxiety items and 7 HADS-depression items. Each item was equipped with a score from 0 to 3. A lower score presents more serious anxiety or depression. Patients with total score ≤ 8 was in a state of anxiety or depression.

PSQI [16] was used to assess the sleep quality of subjects during the latest month. This scale consists of 19 self-assessment and 5 peer assessment items, among which the 19^th^ self-assessment and 5^th^ peer assessment items are not involved in scoring. Here, only the 18 self-assessment items involved in scoring were introduced (see Supplementary material). The 18 items constitute 7 parts, and each part was scored according to 0-3. The cumulative scores of each part were PSQI scores within 0-21. Higher score presents poor sleep quality.

QOL-BREF [17] is a self-management questionnaire with 26 items scored on a 5-point scale. Areas of assessment include physiological field, psychological field, social relationship field, environmental field, subjective feeling of quality of life, subjective feeling of health status, family friction, and appetite. Scores range from 0 to 100. Higher score refers to higher quality of life. Primitive domain scores should be transformed to 0-100 level in convenience of comparison with other datasets. In this transformation, the possible lowest score was set to 0 while the possible highest score was set to 100. Scores among values refer to percentage of total possible scores achieved.

Activity of Daily life Scale (Barthel index) [18] includes 10 items. According to the needs for help and levels of help, the scores were divided into four functional levels (0, 5, 10, 15). The total score is 100. Higher score denotes stronger independence and little dependence.

Health-related life quality was assessed by SGRQ [19], including symptoms, activity, and influence. Total score was considered to be the sum of each domain score. Higher score refers to poor life quality.

The impact of COPD on health status is assessed by CAT [20]. CAT evaluated symptoms like cough, hawking, and dyspnea and the impact of disease on daily life. CAT consists of 8 items, with each item scored within 0-5 (0 = no deficiency). Total score is within 0-40. 0-10, 11-20, 21-30, and 31-40 refer to light, mild, severe, and extremely severe clinical impacts. Lower CAT score denotes higher life quality.

### 2.5. Statistical Analysis

Data were processed by SPSS 25.0 software. The measurement data were first tested for normality. Data that fit normality were displayed as mean value ± standard deviation. Independent sample *t* test was adopted for comparison between two groups. Data that did not fit normality were displayed as IQR. Wilcoxon rank sum test was adopted for comparison among groups. Two category data were denoted as numbers and percentages, and comparison between two groups was chi-square or Fisher exact test. Paired sample *t*-test was used for each score between two groups, scores at admission and after 3 months of discharge in each group. Score change in each group was analyzed by independent sample *t* test. *p* < 0.05 denoted statistical significance.

## 3. Results

### 3.1. Patient's Baseline Characteristics

As shown in Table 1, no significant differences were found with respect to age, sex, body mass index (BMI), disease course, relevant family history, drug allergy history, care level, family average annual income, smoking, profession, payment of medical expense, education degree, marriage status, breathing function exercise history, complications, and medicine of patients in two groups. The two groups were comparable.

### 3.2. Scoring Results

There were no marked differences in anxiety and depression, sleep status, survival quality, Barthel index, SGRQ, and CAT of patients in two groups. After 3 months of discharge, each score was remarkably improved except for anxiety and depression. Moreover, independent sample *t*-test revealed markedly higher improvement of survival quality of patients in the intervention group than that in the control group. As shown in Tables 2 and 3, TC based on a questionnaire survey could conspicuously enhance COPD patient's life quality.

## 4. Discussion

This investigation quantitatively compared the impacts of TC and routine care on disease self-management of COPD patients based on a questionnaire survey. The improvement of survival quality of patients with PT was more significant than patients with routine care. Nevertheless, their anxiety and depression, sleep quality, survival quality, and mobility were not significantly different.

A randomized clinical trial showed that the combination of TC and long-term self-management support lead to more COPD-relevant hospitalizations and emergency visits, without life quality improvement [21]. This study also mentioned that acute care events occur more often in COPD patients who are in a highly activated status when admitted to the hospital. This might trigger inconsistency between its results and ours. In contrast, another study indicated that a website-based TC plan can enhance the life quality and glycemic control of patients with type 2 diabetes after discharge [11]. Likewise, a randomized controlled trial assessed nurse-led TC for old people with open heart surgery. It was suggested that patient's life quality and functional autonomy are markedly increased, while rehospitalization and hospital readmission rates after discharge are conspicuously decreased [22]. TC also improves acute attack patient's life quality [23]. TC conspicuously decreases risk for admission relevant to COPD and caused by COPD [12]. These hold an agreement with our investigation, suggestive of superiority of TC on improving COPD patient's life quality.

Earlier studies presented prevalence of COPD among males than females; therefore, we researched more males. However, with increased smoking of women in high-income countries and more risks for exposure to indoor air pollution (such as biomass fuels for cooking and heating) of women in low-income countries, the future morbidity of COPD among men and women may be equal [24, 25]. Incremental study proved that female is a major risk factor for depression in adult patients with COPD [26]. Of note, intervention time, caregiver type, and telephone visit may affect the outcomes of TC. For example, TC based on app increases self-efficacy of spinal cord injury patients, while it does not improve quality of life significantly [27]. In this investigation, we evaluated COPD patient's life quality via various questionnaires and found positive enhancement. Most previous studies evaluated patient's readmission rates rather than life quality based on various scales. Our investigation presents reliable results in support of the improvement of TC on COPD patients.

However, this investigation has several limitations. First of all, this investigation only included 3-month outcomes of TC based on a questionnaire survey. Thus, the long-term effect, such as readmission rate, was not obtained accurately. Moreover, patient's compliance with drugs should be considered. Secondly, we only collected samples from a hospital, not representative of overall conditions of patients in other regions. COPD morbidity is different in varying areas [28]. Lastly, the sample size in this investigation is small; thus, patient's characteristics themselves may influence the results.

Overall, this investigation confirmed that TC based on a questionnaire survey might improve life quality of COPD patients and perform positive effects on disease self-management. It is necessary to verify the results with more samples and perfect TC programs.

## Figures and Tables

**Table 1:** Baseline characteristics of patients in intervention and control groups.

Characteristics	Intervention group (*n* = 39)	Control group (*n* = 39)	*t*/*χ*^2^/*Z*	*p*
Age, years, median (IQR)	66 (62-68)	64 (59-68)	-1.514	0.130
Sex, *n* (%)				
Male	36 (92.3)	34 (87.2)	0.557	0.711
Female	3 (7.7)	5 (12.8)		
BMI, kg/m^2^, mean (SD)	22.14 ± 3.59	21.24 ± 3.80	1.069	0.289
Disease course, month, median (IQR)	72 (36-120)	64 (21-178)	-0.510	0.610
Relevant family history, *n* (%)	1 (2.6)	0 (0.0)	1.013	1.000
Drug allergy history, *n* (%)	2 (5.1)	1 (2.6)	0.347	1.000
Care level, *n* (%)			1.107	1.000
I	1 (2.6)	0 (0.0)		
II	9 (23.1)	9 (23.1)		
III	27 (69.2)	28 (71.8)		
IV	2 (5.1)	2 (5.1)		
Family average annual income ≤ 50000 CNY, *n* (%)	13 (34.2)	15 (38.5)	0.150	0.814
Profession, *n* (%)			0.217	0.816
Retire	23 (59.0)	25 (64.1)		
Others	16 (41.0)	14 (35.9)		
Current smoking, *n* (%)	25 (64.1)	23 (59.0)	0.217	0.816
Payment of medical expense, *n* (%)			0.963	0.462
Medical insurance	25 (64.1)	29 (74.4)		
Self-expense/partial expense	14 (35.9)	10 (25.6)		
Education degree, *n* (%)			0.396	0.908
Primary school	12 (30.8)	11 (28.2)		
Junior high school	21 (53.8)	20 (51.3)		
Senior high school	6 (15.4)	8 (20.5)		
Marriage status, *n* (%)				
Married	39 (100.00)	39 (100.00)		
Single	0 (0.0)	0 (0.0)		
Breathing function exercise history, *n* (%)	0 (0.0)	1 (2.6)	1.013	1.000
Complications and drugs, *n* (%)	3 (7.7)	2 (5.1)	0.214	1.000
SpO2, median (IQR)	96 (95-97)	96 (95-97)	-0.068	0.946
Hospital day, *d*, median (IQR)	6 (6-8)	7 (6-8)	-0.475	0.635

SpO2: oxyhemoglobin saturation.

**Table 2:** Scoring results of patients in the intervention and control groups.

	Intervention group (*n* = 39)			Control group (*n* = 39)		
	T1	T2	△T	T1	T2	△T
HADS	36.03 ± 4.96	36.85 ± 2.85	0.82 ± 5.34	35.87 ± 5.55	35.79 ± 4.03	−0.08 ± 4.02
PSQI	11.95 ± 9.35	7.87 ± 6.14^∗^	−4.08 ± 7.33	12.23 ± 8.97	10.79 ± 7.83^∗^	−1.44 ± 3.66
QOL-BREF	54.78 ± 8.69	59.74 ± 7.77^∗^	4.96 ± 8.95	54.33 ± 7.92	57.03 ± 8.15^∗^	2.70 ± 6.65
Activity of daily life scale (Barthel index)	76.03 ± 14.79	85.64 ± 16.23^∗^	9.62 ± 23.41	76.28 ± 13.29	79.82 ± 11.66^∗^	3.54 ± 8.86
SGRQ	51.70 ± 19.41	39.45 ± 23.44^∗^	−12.25 ± 24.98	52.87 ± 16.20	47.90 ± 15.00^∗^	−4.97 ± 11.27
CAT	19.85 ± 5.37	14.41 ± 7.51^∗^	−5.44 ± 7.04	20.18 ± 5.07	18.44 ± 5.92^∗^	−1.74 ± 5.29^▲^

T1: on admission; T2: postdischarge third month; △T: pre-post changes; ^∗^*p* < 0.05 compared with before admission in each group, respectively. ^▲^*p* < 0.05 compared with changes in the control group.

**Table 3:** *p* values of two groups after scale scoring.

	Intervention group (*n* = 39)	Control group (*n* = 39)	*p* value (differences between groups after treatment)
	*p* values before and after treatment	*p* values before and after treatment	
HADS	0.343	0.906	0.404
PSQI	0.001	0.019	0.048
QOL-BREF	0.001	0.016	0.210
Activity of daily life scale (Barthel index)	0.014	0.017	0.134
SGRQ	0.004	0.009	0.101
CAT	<0.001	0.046	0.011

## Data Availability

The data used to support the findings of this study are included within the article.

## Supplementary Materials

Supplementary Materials I. Hospital Anxiety and Depression Scale (HADS)
