# Supplementary material for: Questionnaire Survey-Based Quantitative Assessment of the Impact of Transitional Care on Self-Management of Patients with Acute Exacerbation of Chronic Obstructive Pulmonary Disease
Source: Comput Math Methods Med. 2021 Nov 13;2021:3634548. doi: 10.1155/2021/3634548 (PMC8605913; doi:10.1155/2021/3634548)
Supplement: Supplementary Materials — I. Hospital Anxiety and Depression Scale (HADS) [file 3634548.f1.doc]

I. Hospital Anxiety and Depression Scale (HADS)

Please read each item and chose the reply which comes closest to how you have been feeling in the last month. Do not take too long over your replies. Your immediate reaction to each item will probably be more accurate than a long thought-out response. There is no true or false answer. Information you provided is used for medical research only and will be completely confidential. Thanks for your cooperation!

1．I fell tense or “wound up”:

a. most of the time b. a lot of the time c. from time to time, occasionally d. not at all.

2．I still enjoy the things I used to enjoy:

a. Definitely as much b. not quite so much c. only a little d. hardly at all

3．I get a sort of frightened feeling as if something awful is about to happen:

a. very definitely and quite badly b. yes, but not too badly c. a little, but it doesn’t worry me d. not at all

4．I can laugh and see the funny side of things:

a. As much as I always could b. Not quite so much now c. Definitely not so much now d. Not at all

5．Worrying thoughts go through my mind:

a. a great deal of the time b. a lot of the time c. from time to time, but not too often d. only occasionally

6．I feel cheerful:

a. not at all b. not often c. sometimes d. most of the time

7．I can sit at ease and feel relaxed:

a. definitely b. usually c. not often d. not at all

8．I have lost interest in my appearance:

a. definitely b. I don’t take as much care as I should c. I may not take quite as much care d. I take just as much care as ever

9．I feel restless as I have to be on the move:

a. very much indeed b. quite a lot c. not very much d. not at all

10. I look forward with enjoyment to things:

a. as much as I ever did b. rather less than I used to c. definitely less than I used to d. hardly at all

11. I get sudden feelings of panic:

a. very often indeed b. quite often c. not very often d. not at all

12. I feel as if I am slowed down:

a. nearly all the time b. very often c. sometimes d. not at all

13. I get a sort of frightened feeling like “butterflies” in the stomach:

a. not at all b. occasionally c. quite often d. very often

14. I can enjoy a good book or radio or tv program

a. often b. sometimes c. not often d. very seldom

Ⅱ. Pittsburgh Sleep Quality Index (PSQI)

Instructions: the following questions relate to your usual sleep habits during the past month only. Your answers should indicate the most accurate reply for the majority of days and rights in the past month. The information you provided is for medical research purpose only and will be completely confidential. Thanks for your cooperation!

1. During the past month, what time have you usually gone to bed at night? ___________________

2. During the past month, how long (in minutes) has it usually taken you to fall asleep each night? __________

3. During the past month, what time have you usually gotten up in the morning? ___________________

4. During the past month, how many hours of actual sleep did you get at night? (This may be different than the number of hours you spent in bed.) ___________________

5. During the past month, how often have you had trouble sleeping because you…

a. Cannot go to sleep within 30 minutes

(1) Not during the past month (2) Less than once a week (3) Once or twice a week (4) Three or more times a week

b. Wake up in the middle of the night or early morning

(1) Not during the past month (2) Less than once a week (3) Once or twice a week (4) Three or more times a week

c. Have to get up to use the bathroom

(1) Not during the past month (2) Less than once a week (3) Once or twice a week (4) Three or more times a week

d. Cannot breathe comfortably

(1) Not during the past month (2) Less than once a week (3) Once or twice a week (4) Three or more times a week

e. Cough or snore loudly

(1) Not during the past month (2) Less than once a week (3) Once or twice a week (4) Three or more times a week

f. Feel too cold

(1) Not during the past month (2) Less than once a week (3) Once or twice a week (4) Three or more times a week

g. Feel too hot

(1) Not during the past month (2) Less than once a week (3) Once or twice a week (4) Three or more times a week

h. Had bad dreams

(1) Not during the past month (2) Less than once a week (3) Once or twice a week (4) Three or more times a week

i. Have pain

(1) Not during the past month (2) Less than once a week (3) Once or twice a week (4) Three or more times a week

j. Other reason(s), please describe___________________ How often during the past month have you had trouble sleeping because of this?

(1) Not during the past month (2) Less than once a week (3) Once or twice a week (4) Three or more times a week

6. During the past month, how would you rate your sleep quality overall?

a. Very good b. Fairly good c. Fairly bad d. Very bad

7. During the past month, how often have you taken medicine to help you sleep?

a. Not during the past month b. Less than once a week c. Once or twice a week d. Three or more times a week

8. During the past month, how often have you had trouble staying awake?

a. Not during the past month b. Less than once a week c. Once or twice a week d. Three or more times a week

9. During the past month, how often do your feel tired?

a. None b. Occasionally c. Sometimes d. Often

Ⅲ. QOL-BREF

The following questions ask how you feel about your quality of life, health, or other areas of

your life. I will read out each question to you, along with the response options. Please choose the answer that appears most appropriate. If you are unsure about which response to give to a question, the first response you think of is often the best one.

Please keep in mind your standards, hopes, pleasures and concerns. We ask that you think

about your life in the last two weeks.

For example: Could you get support you need from others?

| Not at all | A little | Moderately | Mostly | completely |
| --- | --- | --- | --- | --- |
| 1 | 2 | 3 | 4 | 5 |

Please tick a √ at the most appropriate number based on how much support you have received from others over the past two weeks, a √ at “4” if you get the support you need most of the time, and a √ at “1” if you don't get the support you need at all.

Please read each question and chose the most appropriate answer.

1. (G1) How would you rate your quality of life?

| Very poor | poor | Neither poor nor good | good | Very good |
| --- | --- | --- | --- | --- |
| 1 | 2 | 3 | 4 | 5 |

2. (G4) How satisfied are you with your health?

| Very dissatisfied | Dissatisfied | Neither  satisfied nor  dissatisfied | Satisfied | Very satisfied |
| --- | --- | --- | --- | --- |
| 1 | 2 | 3 | 4 | 5 |

The following questions ask about how much you have experienced certain things in the last two weeks.

3. (F1.4) To what extent do you feel that physical pain prevents you from doing what you need to do?

| Not at all | A little | A moderate amount | Very much | An extreme amount |
| --- | --- | --- | --- | --- |
| 1 | 2 | 3 | 4 | 5 |

4. (F11.3) How much do you need any medical treatment to function in your daily life?

| Not at all | A little | A moderate amount | Very much | An extreme amount |
| --- | --- | --- | --- | --- |
| 1 | 2 | 3 | 4 | 5 |

5. (F4.1) How much do you enjoy life?

| Not at all | A little | A moderate amount | Very much | An extreme amount |
| --- | --- | --- | --- | --- |
| 1 | 2 | 3 | 4 | 5 |

6. (F24.2) To what extent do you feel your life to be meaningful?

| Not at all | A little | A moderate amount | Very much | An extreme amount |
| --- | --- | --- | --- | --- |
| 1 | 2 | 3 | 4 | 5 |

7. (F5.3) How well are you able to concentrate?

| Not at all | A little | A moderate amount | Very much | An extreme amount |
| --- | --- | --- | --- | --- |
| 1 | 2 | 3 | 4 | 5 |

8. (F16.1) How safe do you feel in your daily life?

| Not at all | A little | A moderate amount | Very much | An extreme amount |
| --- | --- | --- | --- | --- |
| 1 | 2 | 3 | 4 | 5 |

9. (F22.1) How healthy is your physical environment?

| Not at all | A little | A moderate amount | Very much | An extreme amount |
| --- | --- | --- | --- | --- |
| 1 | 2 | 3 | 4 | 5 |

The following questions ask about how completely you experience or were able to do certain things in the last two weeks.

10. (F2.1) Do you have enough energy for everyday life?

| Not at all | A little | moderately | mostly | completely |
| --- | --- | --- | --- | --- |
| 1 | 2 | 3 | 4 | 5 |

11. (F7.1) Are you able to accept your bodily appearance?

| Not at all | A little | moderately | mostly | completely |
| --- | --- | --- | --- | --- |
| 1 | 2 | 3 | 4 | 5 |

12. (F18.1) Have you enough money to meet your needs?

| Not at all | A little | moderately | mostly | completely |
| --- | --- | --- | --- | --- |
| 1 | 2 | 3 | 4 | 5 |

13. (F20.1) How available to you is the information that you need in your day-to-day life?

| Not at all | A little | moderately | mostly | completely |
| --- | --- | --- | --- | --- |
| 1 | 2 | 3 | 4 | 5 |

14. (F21.1) To what extent do you have the opportunity for leisure activities?

| Not at all | A little | moderately | mostly | completely |
| --- | --- | --- | --- | --- |
| 1 | 2 | 3 | 4 | 5 |

15. (F9.1) How well are you able to get around?

| Very poor | poor | Neither poor nor good | good | Very good |
| --- | --- | --- | --- | --- |
| 1 | 2 | 3 | 4 | 5 |

The following questions ask about how you feel in every aspect of your daily life in the last four weeks.

16. (F3.3) How satisfied are you with your sleep?

| Very dissatisfied | dissatisfied | Neither satisfied nor dissatisfied | satisfied | Very satisfied |
| --- | --- | --- | --- | --- |
| 1 | 2 | 3 | 4 | 5 |

17. (F10.3) How satisfied are you with your ability to perform your daily living activities?

| Very dissatisfied | dissatisfied | Neither satisfied nor dissatisfied | satisfied | Very satisfied |
| --- | --- | --- | --- | --- |
| 1 | 2 | 3 | 4 | 5 |

18. (F12.4) How satisfied are you with your capacity for work?

| Very dissatisfied | dissatisfied | Neither satisfied nor dissatisfied | satisfied | Very satisfied |
| --- | --- | --- | --- | --- |
| 1 | 2 | 3 | 4 | 5 |

19. (F6.3) How satisfied are you with yourself?

| Very dissatisfied | dissatisfied | Neither satisfied nor dissatisfied | satisfied | Very satisfied |
| --- | --- | --- | --- | --- |
| 1 | 2 | 3 | 4 | 5 |

20. (F13.3) How satisfied are you with your personal relationships?

| Very dissatisfied | dissatisfied | Neither satisfied nor dissatisfied | satisfied | Very satisfied |
| --- | --- | --- | --- | --- |
| 1 | 2 | 3 | 4 | 5 |

21. (F15.3) How satisfied are you with your sex life?

| Very dissatisfied | dissatisfied | Neither satisfied nor dissatisfied | satisfied | Very satisfied |
| --- | --- | --- | --- | --- |
| 1 | 2 | 3 | 4 | 5 |

22. (F14.4) How satisfied are you with the support you get from your friends?

| Very dissatisfied | dissatisfied | Neither satisfied nor dissatisfied | satisfied | Very satisfied |
| --- | --- | --- | --- | --- |
| 1 | 2 | 3 | 4 | 5 |

23. (F17.3) How satisfied are you with the conditions of your living place?

| Very dissatisfied | dissatisfied | Neither satisfied nor dissatisfied | satisfied | Very satisfied |
| --- | --- | --- | --- | --- |
| 1 | 2 | 3 | 4 | 5 |

24. (F19.3) How satisfied are you with your access to health services?

| Very dissatisfied | dissatisfied | Neither satisfied nor dissatisfied | satisfied | Very satisfied |
| --- | --- | --- | --- | --- |
| 1 | 2 | 3 | 4 | 5 |

25. (F23.3) How satisfied are you with your transport?

| Very dissatisfied | dissatisfied | Neither satisfied nor dissatisfied | satisfied | Very satisfied |
| --- | --- | --- | --- | --- |
| 1 | 2 | 3 | 4 | 5 |

The following question refers to how often you have felt or experienced certain things in the last four weeks.

26. (F8.1) How often do you have negative feelings such as blue mood, despair, anxiety, depression?

| never | seldom | Quite often | Very often | always |
| --- | --- | --- | --- | --- |
| 1 | 2 | 3 | 4 | 5 |

Besides, there are three questions.

101. Does family friction affect your life?

| Not at all | A little | moderately | mostly | completely |
| --- | --- | --- | --- | --- |
| 1 | 2 | 3 | 4 | 5 |

102. How is your appetite?

| Very poor | poor | Neither poor nor good | good | Very good |
| --- | --- | --- | --- | --- |
| 1 | 2 | 3 | 4 | 5 |

103. How will you rate your overall quality of life (including biological health, psychological health, social relationship and surrounding environment, etc.)? ____________ (total score: 100).

Do you fill this questionnaire with someone’s help? Yes No

How long do you spend to fill this questionnaire? ( ) min (s)

Ⅳ. Activities of Daily Life (Barthel index)

| Item | Score | Standard | Assessment date | | |
| --- | --- | --- | --- | --- | --- |
|  |  |  |
| Bowels | 0  5  10 | Incontinent (or needs to be given enemata)  Occasional accident (once/week)  continent |  |  |  |
| Bladder | 0  5  10 | Incontinent, or catheterized and unable to manage  Occasional accident (max once per 24 hours)  continent |  |  |  |
| Grooming | 0  5 | Needs help with personal care  Independent face/hair/teeth/shaving (implements provided) |  |  |  |
| Toilet use | 0  5  10 | dependent  needs some help, but can do something alone  independent (on and off, dressing, wiping) |  |  |  |
| Feeding | 0  5  10 | Unable or completely dependent  Needs help cutting, spreading butter, etc.  Independent (food provided within reach) |  |  |  |
| Transfer | 0  5  10  15 | Unable – no sitting balance  Major help (one or two people, physical), can sit  Minor help (verbal or physical)  Independent |  |  |  |
| Mobility | 0  5  10  15 | immobile  wheelchair independent, including corners, etc.  walks with help of one person (verbal or physical)  independent (but may use any aid, e.g., stick) |  |  |  |
| Dressing | 0  5  10 | Dependent  Needs help, but can do about half unaided  Independent (including buttons, zips, laces, etc.) |  |  |  |
| Stairs | 0  5  10 | unable  needs help (verbal, physical, carrying aid)  independent up and down |  |  |  |
| Bathing | 0  5 | dependent  independent (or in shower) |  |  |  |
| Total score | | |  |  |  |
| Evaluator | | |  |  |  |

Ⅴ. ST. GEORGE’S RESPIRATORY QUESTIONNAIRE (SGP.Q)

This questionnaire is designed to help us learn much more about how your breathing is troubling you and how it affects your life. We are using it to find out which aspects of your illness cause you most problems, rather than what the doctors and nurses think your problems are. Please ask if you have difficulty understanding the questions. Do not spend too long deciding about your answers.

**PART 1**

1. During the past one year, how often do you cough?

(1) Most days a week

(2) Several days a week

(3) A few days a month

(4) Only with respiratory infections

(5) Not at all

2. During the past one year, how often do you bring up phlegm (sputum)?

(1) Most days a week

(2) Several days a week

(3) A few days a month

(4) Only with respiratory infections

(5) Not at all

3. During the past four weeks, how often do you have shortness of breath?

(1) Most days a week

(2) Several days a week

(3) A few days a month

(4) Only with respiratory infections

(5) Not at all

4. During the past four weeks, how often do you have attacks of wheezing?

(1) Most days a week

(2) Several days a week

(3) A few days a month

(4) Only with respiratory infections

(5) Not at all

5. During the past four weeks, how many breathing difficulties did you have during the last year?

(1) More than 3 attacks

(2) 3 attacks

(3) 2 attacks

(4) 1 attack

(5) None

6. How long does the most severe episode of breathing difficulties last? (If there is no severe episode, skip to question 7.)?

(1) 1 week or more

(2) 3 days or more

(3) 1-2 days

(4) Less than 1 day

7. How often do you have good days (with few respiratory problems)?

(1) No good days

(2) 1-2 good days

(3) 3-4 good days

(4) Most days are good

(5) Every day is good

8. If you have a wheeze, is it worse when you get up in the morning? If you don’t have a wheeze, directly skip to part II.

□ No □ Yes

**Part II**

9. How would you describe your respiratory problems?

(1) The most serious problems

(2) A lot of problems

(3) A few problems

(5) No problems

10. If you have worked, select one of the following answers.

(1) Breathing problems terminate my work

(2) Breathing problems affect or change my work

(3) breathing problems don’t affect my work

11. Questions about what activities usually make you feel breathless. For each statement, please tell me which applies to you these days.

Yes No

Sitting or lying quietly □ □ Washing or dressing yourself □ □ Walking around the house □ □ Walking outside on the level ground □ □ Walking up a flight of stairs □ □ Walking up hills □ □ Sports activities or games □ □

12．Some more questions about your cough and breathlessness. For each statement, please tell me which applies to you these days.

Yes No

Coughing hurts □ □ Coughing makes me tired □ □

I am short of breath when I talk □ □

I am short of breath when I bend over □ □ My cough or breathing disturbs my sleep □ □

I get exhausted easily □ □

13．Questions about other effects that your chest trouble may have on you. For each statement, please tell me which applies to you these days.

Yes No

My cough or breathing is embarrassing in public □ □ A nuisance to my family, friends, or neighbors □ □

I get afraid or panic when I cannot catch my breath □ □

I feel that I am not in control of my respiratory problems □ □

I don't expect my breathing problem to get better □ □

I have become frail or an invalid because of my respiratory problems □ □ Exercise is not safe for me □ □ Everything seems too much of an effort □ □

14. Questions about your treatment conditions. If you don’t have a treatment, please skip this question. For each statement, please tell me which applies to you these days.

Yes No My treatment is not of great significance □ □ I was embarrassed to take drugs in front of others □ □ My treatment leaves me adverse drug effects □ □ My treatment interferes my life □ □

15. These are questions about how your activities might be affected by your respiratory problems. For each statement, please tell me which applies to you because of your breathing.

Yes No

I take a long time to get washed or dressed □ □

I cannot take a bath or shower, or I take a long time to do it □ □

I walk slower than other people, or I stop to rest □ □

Jobs such as house chores take a long time, or I have to stop to rest □ □

If I walk up one flight of stairs, I have to go slowly or stop □ □

If I hurry or walk fast, I have to stop or slow down □ □

My breathing makes it difficult to walk up hills, carry things up stairs, do light gardening such as weeding, dance, bowl, or play golf □ □

My breathing makes it difficult to do things such as carry heavy loads, dig the garden or shovel snow, jog or walk briskly (5 miles per hour), play tennis, or swim □ □

My breathing condition makes it difficult for me to perform tasks such as heavy physical labor, running, cycling, swimming at a fast pace, or engaging in vigorous physical activity □ □

16. We would like to know how your chest usually affects your daily life. For each statement, please tell me which applies to you because of your breathing.

Yes No

I cannot play sports or do other physical activities □ □

I cannot go out for entertainment or recreation □ □

I cannot do household chores □ □

I cannot move far from my bed or chair □ □

Here are some other activities you may not be able to do due to your breathing problems. (You don't have to choose yes or no, they just remind you what asthma can do to you.)

walk

Do household chores

Have sex

Go out in bad weather or enter a room that smells a smoke

Visit relatives or friends and play with kids

Please write down below any other important activities that you are unable to perform due to your breathing problems:

17. How do your respiratory problems affect you? Please pick one response

(1) They do not stop me from doing anything I would like to do

(2) They stop me from doing one or two things I would like to do

(3) They stop me from doing most of the things I would like to do

(4) They stop me from doing everything I would like to do

Ⅵ. COPD Assessment Test (CAT)

| Instructions: for the following items, please fill the most appropriate number corresponding to your current status in “at admission” and “after discharge”. For example: ①②③④⑤, I am very happy, I am very unhappy | | | | |
| --- | --- | --- | --- | --- |
|  | | | At admission | After 3 months of discharge |
| I never cough | ①②③④⑤ | I cough all the time |  |  |
| I have no phlegm (mucus) in my chest at all | ①②③④⑤ | My chest is completely full of phlegm (mucus) |  |  |
| My chest does not feel tight at all | ①②③④⑤ | My chest feels very tight |  |  |
| When I walk up a hill or one flight of stairs I am not breathless | ①②③④⑤ | When I walk up a hill or one flight of stairs I am very breathless |  |  |
| I am not limited doing any activities at home | ①②③④⑤ | I am very limited doing activities at home |  |  |
| I am confident leaving my home despite my condition | ①②③④⑤ | I am not at all confident leaving my home because of my lung condition |  |  |
| I sleep soundly | ①②③④⑤ | I don’t sleep soundly because of my lung condition |  |  |
| I have lots of energy | ①②③④⑤ | I have no energy at all |  |  |
| Total score | | |  |  |
